# Shear Bond Strength of Ah26 to Human Dentin Treated with Dimethyl Sulfoxide (Dmso)

**DOI:** 10.3390/dj10060100

**Published:** 2022-06-06

**Authors:** Fotis Dimopoulos, Konstantinos Kodonas, Christos Gogos

**Affiliations:** Department of Endodontology, School of Dentistry, Aristotle University of Thessaloniki, 54124 Thessaloniki, Greece; kkodonas@gmail.com (K.K.); gogos@dent.auth.gr (C.G.)

**Keywords:** dentistry, bond strength, sealer, AH26, dimethyl sulfoxide

## Abstract

The purpose of this study was to examine the bond strength of AH26 to human coronal dentin exposed to DMSO. A total of 70 dentin specimens were equally divided into two groups. Each dentin surface was pre-treated with 2 mL of 2.5% NaOCl, 3 mL of EDTA 17%, and distilled water. One group was finally rinsed with 50% DMSO. Following the AH26 application, the bond strength was tested by subjecting the samples to a shear load at a crosshead speed of 0.5 mm/min using universal testing equipment. The results according to paired samples *t*-test indicated that there was a statistically insignificant difference between the two groups. Therefore, DMSO had no effect on the bond strength of AH26 root sealer to dentin.

## 1. Introduction

A tight seal of the root filling material against bacteria is one significant key to achieving a long-term successful endodontic treatment. Gutta-percha is the most commonly used obturating material [1]. Since gutta-percha does not impulsively bond to dentin walls, the use of a root canal sealer to enhance the core filling material adaptation is of considerable importance [1]. In this way, bond failure of endodontic sealers remains a clinical problem and a possible cause of unsuccessful endodontic treatment [2,3]. Therefore, an endodontic sealer should adhere firmly to both dentin and gutta-percha [3]. Adhesion appears desirable for two reasons. In a static situation, it eliminates any space that allows for the penetration of fluids between the gutta-percha and the canal wall. In a dynamic situation, it resists any tendency toward dislodgment of the filling following manipulation, e.g., after space preparation [4].

An appropriate bond between the filling material and the canal walls can be achieved either chemically or via micromechanical retention. The quality of the bond affects the resisting dislodgment of the filling material of the root canal and maintains the integrity of the crucial core–dentin interface. Epoxy resin-based sealers have proven their ability in this prospect in various studies [5,6,7,8,9]. They are characterized by a reactive epoxide ring that breaks after polymerization, establishing a molecular bond with the dentin surface. Besides molecular attraction, the bond strength is enhanced by mechanical interlocking following the penetration of the material to dentinal tubules and irregularities on the dentin surface [10]. Therefore, the sealer used in this study was AH26.

As a polar aprotic solvent with polar and non-polar elements, dimethyl sulfoxide (DMSO; (CH_3_)_2_SO) is a highly polar aprotic solvent with a polar S=O group and two hydrophobic CH_3_ groups [11]. This solvent is miscible in solvents and most adhesive monomers and is considered to be one of the best currently used penetration enhancers for medical purposes due to its amphiphilic nature, small size, and dipolar aprotic nature [12]. Practically, in the field of adhesive dentistry, DMSO’s ability to separate the highly cross-linked collagen into a sporadic network of apparent fibrils in the dentin matrix by suppression of hydrogen-bond-mediated attractive forces within collagen is considered essential for increasing the bond strength of the material to dentin [13]. Numerous studies showed that acid-etched dentin treated with dimethyl sulfoxide may improve long-term bond conservation via the inhibition of collagenolytic enzymes [12] and increase the immediate bond strength with an etch-and-rinse adhesive as a result of the improved penetration of adhesive into the exposed collagen matrix [13].

Previous studies showed that a DMSO wet bonding technique is effective to increase collagen incapsulation and improve the resin–dentin bond quality [12,13,14]. A 50% DMSO concentration was an attainable alternative to enhance resin–dentin bond quality, especially for water-based etch-and-rinse adhesive systems. Similar findings for the improved bond strength of adhesive systems using DMSO were reported in other studies [13,14]. In contrast, there is no research to examine the effect of DMSO on the adhesion ability of endodontic sealers to dentin. Considering DMSO’s main ability to dissociate the highly cross-linked collagen into a sporadic network of apparent fibrils in a dentin matrix, this study investigated whether this matrix of divided collagen may be beneficial for the bonding of epoxy-resin-based sealer and dentin, as their epoxide rings bond chemically to the amino groups of dentinal collagen [15].

The purpose of the present study was to examine the bond strength of AH26 to human coronal dentin exposed to DMSO. The null hypothesis was that the dentin pre-treatment with DMSO enhances the bond strength of AH26 root sealer to dentin.

## 2. Materials and Methods

The study was conducted in accordance with the Declaration of Helsinki ethical standards and the protocol was approved by the local institutional review board and ethics committee (protocol no./date. 107/ 1 February 2022).

The materials used were AH26 sealer (AH26, Topseal; Dentsply, Konstanz, Switzerland) and dimethyl sulfoxide (DMSO) (Table 1).

### 2.1. Specimen Preparation

**Scheme 1:**
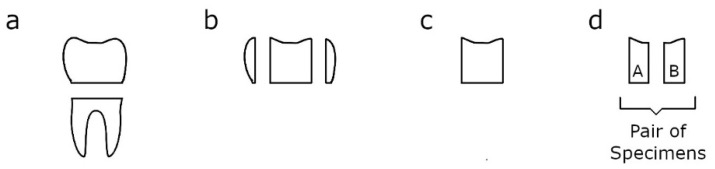
Specimen preparation ((**a**) section below the cementoenamel junction (**b**) longitudinally section in the buccolingual direction from both the mesial and distal surface (**c**) remaining specimen (**d**) section in the middle of the remaining specimen resulting to two equal parts).

A total of 35 mandibular third molar human teeth from 18 25-year-old patients extracted within 4 months were used in this study. Teeth were obtained after patient informed consent was given under a protocol approved by the Ethical Committee of the Aristotle University of Thessaloniki Dental School. All selected teeth had no caries, were free of any developmental defects, had no visible cracks, and had not previously undergone any dental restoration. Teeth were stored in 0.2% thymol disinfectant (Mallinckrodt Baker, Phillipsburg, NJ, USA) at 4 °C [16]. All teeth were scaled with an ultrasonic scaler tip (EMS Electro Medical Systems SA, Nyon, Switzerland) to deduct calculus and remnants of the periodontal ligament, washed under running tap water to eliminate thymol residues, and dried with absorbent paper. The teeth were sectioned below the cementoenamel junction with a diamond wafering blade (Isomet Blade, Buehler, Waukegan, IL, USA). The crown was then split longitudinally in the buccolingual direction from both the mesial and distal surface of the teeth using a low-speed diamond disk saw with water coolant (Buehler^®^ Isomet™ low-speed saw). The remaining specimen was then cut into two equal parts and the outer mesial and distal surfaces of each specimen were wet-polished until it was smooth and flat using 600-grit silicon carbide paper (Scheme 1). Every specimen was pre-treated with 2 mL of 2.5% NaOCl, 17% EDTA solution, and distilled water.

**Figure 1:**
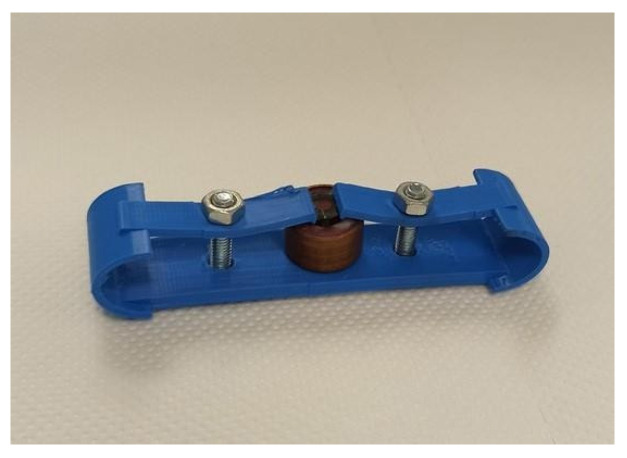
Modified stationary clamp for plastic cylinder stabilization.

**Figure 2:**
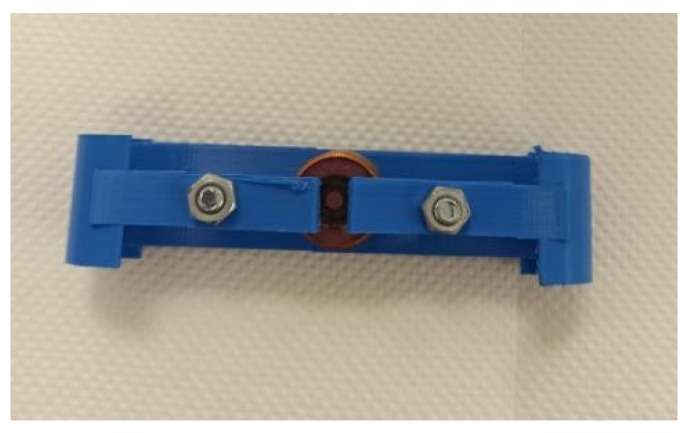
Modified stationary clamp for plastic cylinder stabilization.

Each dentin sample was embedded in a copper ring resting on a glass slab. A light-cured acrylic material was used to fill the remaining volume of the ring to secure the specimens. Modified stationary clamps were used to stabilize the plastic cylinders (height 2 mm, diameter 2 mm) over a substrate and standardize the bonding area (Figure 1 and Figure 2).

Two samples were obtained from each tooth, with the first specimen allocated into group A and the second to group B until all dentin specimens were randomly divided into 2 equal groups (A and B) of 35 specimens each. Dentin samples of groups A and B were treated using two different protocols. More specifically, in group A, the dentin specimens were rinsed with 3 mL EDTA 17% (Largal Ultra, Septodont, France) for 2 min, rinsed with distilled water for 10 s, and then dried with an air spray for 10 s before sealer application. AH26 sealer was applied with a hand plugger (Buchanan Hand Plugger No. 2, Kerr Endodontics). The sealer was mixed according to the manufacturer’s instructions (powder/liquid ration 2:1). In group B, dentin was etched for 2 min with 3 mL 17% EDTA and then rinsed with distilled water for 10 s. The substrate was dried with an air spray for 10 s, followed by an active application of 50% DMSO for 60 s using a disposable cavity brush. DMSO concentration and application time were set accordingly to avoid the complete disruption of dentin collagen. The dentin was then gently air-dried for 5 s and, finally, AH26 sealer was applied using a hand plugger as in group A. The groups, dentin treatments, and materials are summarized in Table 2.

After placement of the endodontic sealer, all samples, comprising dentin specimens and bonded materials, were left for 4 h at room temperature and then placed in an incubator (37 °C, 100% humidity) for 1 week. The samples were then subjected to shear bond strength testing and further assessed under a stereomicroscope to evaluate the failure mode.

### 2.2. Shear Bond Strength Testing

Every sample was loaded parallel to the adhesive interface that formed between the dentin and test material. The measurement of bond strength was conducted with a universal testing machine (UltratesterTM, Ultradent, South Jordan, UT, USA), which subjected specimens to a shear load at a crosshead speed of 0.5 mm/min using recommended parameters for testing [10]. The required force to break the bond between the sealer and dentin was recorded in kg. The measurement was conducted using a special device Sentan kb3 (Sentran, LLC 4355NLoweell street, Ontario, CA, USA) connected to the testing machine. The megapascal (MPa) was the measurement unit for the shear strength of the bond.

After the shear bond testing, all samples were inspected with a stereomicroscope (Nikon SMZ10, Tokyo, Japan) at 20× magnification to determine the failure mode. The failure mode was recorded as adhesive when bonding failure was observed at the substrate–adhesive interface; cohesive when debonding was observed within the adhesive material not involving the interface; or mixed (cohesive and adhesive), which involved debonding within the adhesive material and at the interface [10].

### 2.3. Statistics

The sample size for the present study was determined from a previous pilot study in which an effect size of 0.5 was added to a power (1−b)=0.95 and a level of significance equal to a=0.05. The data were analyzed into a procedure of a t-test family (paired-samples *t*-test) with G*power 3.1 for Windows software (G power 3.1.9.2, program written by Franz Faul, Kiel University, Kiel, German) [10,13]. The optimal sample size was calculated to be more than 35 statistical pairs.

In the first step of the analysis, the data were examined for normal distribution with the Shapiro–Wilk test, as well as the complementary Kolmogorov–Smirnov test. Both tests provide evidence in favor of a normal distribution (p > 0.05). After providing evidence of a normal distribution, the paired-samples t-test was used in order to examine statistically significant differences between groups. For the needs of the analysis, the IBM SPPS version 25 software package was used and all tests were provided under a 5% level of significance.

## 3. Results

The mean shear bond strengths in MPa and standard deviations are presented in Table 3. The results indicate similar values between the two groups.

The results of the paired samples *t*-test indicated a statistically insignificant difference between variables, while the probability value of the test was found to be above the critical level of 5% (*p* > 0.05) (Table 4).

**Figure 3:**
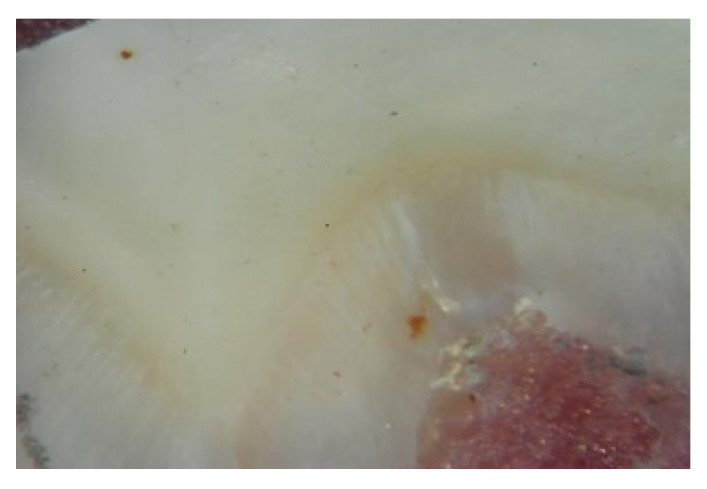
Adhesive failure—group A.

**Figure 4:**
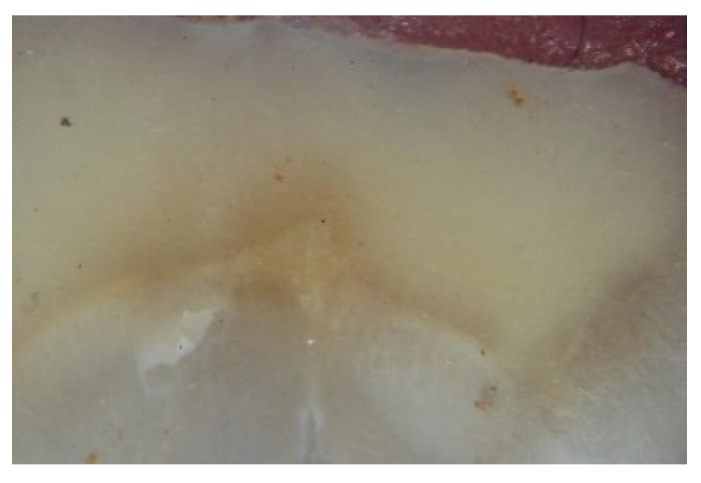
Adhesive failure—group B.

**Figure 5:**
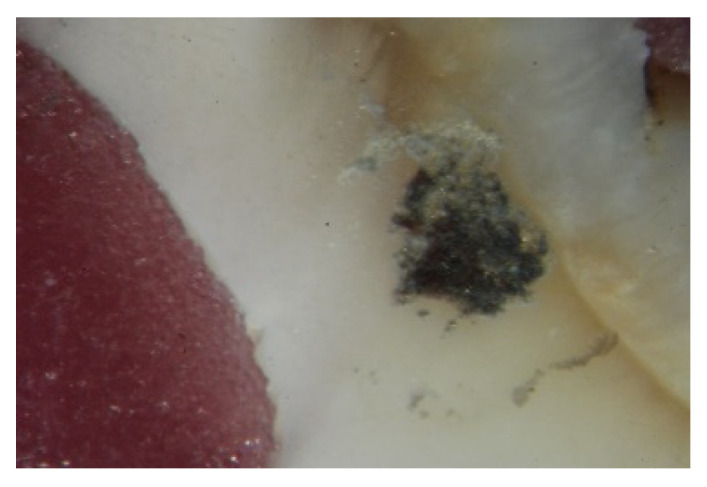
Cohesive failure—group A.

**Figure 6:**
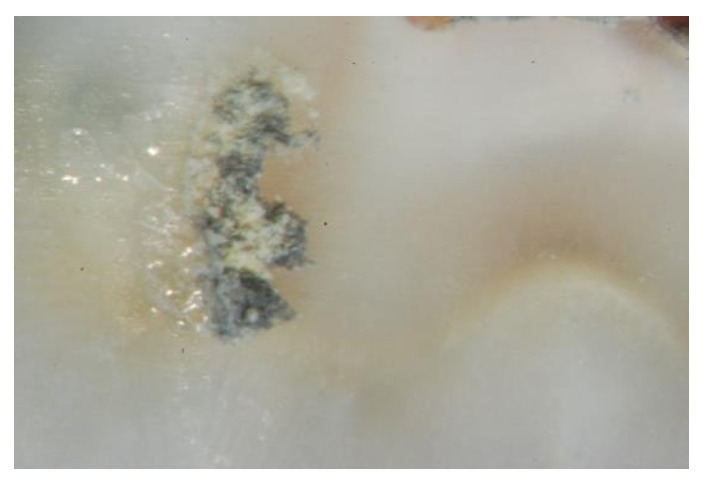
Cohesive failure—group B.

**Figure 7:**
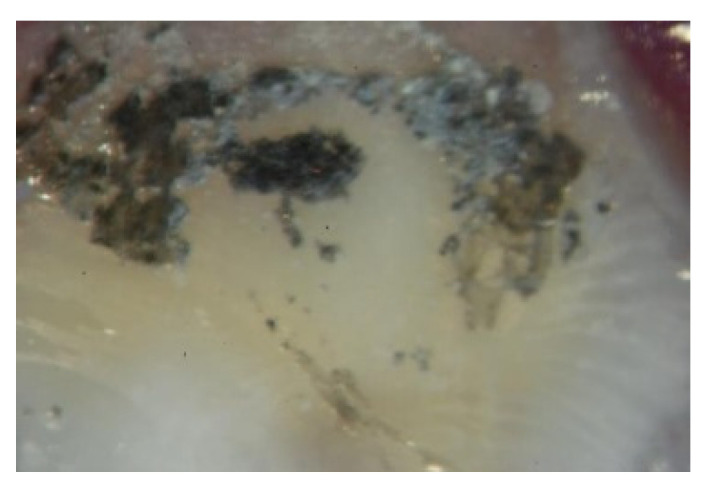
Mixed failure—group A.

**Figure 8:**
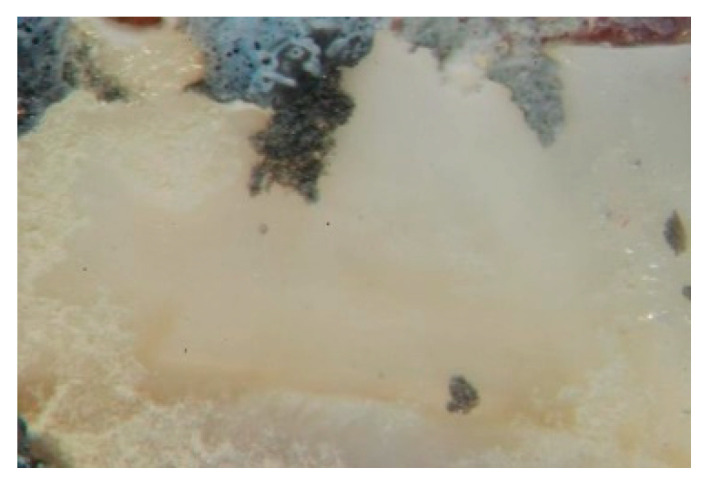
Mixed failure—group B.

Microscopic examination of all fractured surfaces revealed the bond failure to be mainly adhesive for the AH26 sealer without DMSO (group A). AH26 without DMSO (group A) showed 77.1% adhesive, 14.3% cohesive, and 8.6% mixed failures. AH26 with DMSO (group B) showed 71.5% adhesive, 17.1% cohesive, and 11.4% mixed failures. The confidence interval was calculated to be 95%. The failure patterns of the bonds between the two groups are summarized in Table 5. Images from every failure model were obtained during microscopic examination (Figure 3, Figure 4, Figure 5, Figure 6, Figure 7 and Figure 8).

## 4. Discussion

A tight seal against bacteria is one of the fundamental goals of root canal obturation in endodontics. For this purpose, research efforts are concentrated on the search for an ideal sealer with improved properties, such as adhesion and tubular penetration, and the ideal method of dentin pretreatment to enhance sealer adaptation. The pre-treatment of dentin with 50% DMSO has been widely investigated in adhesive dentistry. So far, it is evident that this solvent possesses the ability to separate the fully cross-linked collagen into a sporadic network of separate fibrils in a dentin matrix by subduing the hydrogen-bond-mediated attractive forces within collagen [17]. DMSO also has the ability of dentin surface free energy suppression, which improves the wettability and adhesive penetration. Consequently, the potential use of DMSO for the improvement of sealer adhesion to dentin may be of some importance presenting a ground base for further investigation. In this study, the utilization of all DMSO’s properties was the motivation to use this solvent in order to produce a tight and persistent interface between the root canal sealer and the dentin [18]. The null hypothesis that the dentin pretreatment with DMSO enhances the bond strength of AH26 root sealer to dentin was rejected because DMSO had no substantial effect on the bond strength of AH26 root sealer to dentin.

Smear layer creation on hard tissues is conducted when cuts are made by utilizing hand or rotary instruments. As indicated by Eldarrata et al. [19], the smear layer thickness is approximately 0.5 µm and it forms on the dentine surface by organic components such as hydroxyapatite, saliva, blood, and bacteria. The composition of the smear layer consists of two amorphous coatings: a superficial layer and a deep layer. The extension of a deep layer can be up to 110 µm within the dentinal tubules. It is called a smear plug. A smear layer seals the adhesive interface and does inhibit adhesive–dentin bonding. Consequently, it has been widely accepted that dentin conditioning with a chelating agent improves sealer adhesion properties [20]. In this study, dentin samples were etched with 17% EDTA to remove the smear layer and achieve better adhesion. Removing the smear layer is considered essential since it allows for better cleaning and disinfecting of the root canal walls but also for the activation of adhesion mechanisms and better adaptation of root canal filling materials. The two distinguished adhesion mechanisms involved include chemical and mechanical adhesion of the sealer to dentin. A smooth dentin surface results in better chemical adhesion. On the other hand, micromechanical bonding can be achieved by the penetration of the material to dentin matrix irregularities and dentinal tubules. Consequently, it is evident that smear layer removal assists in the bonding between dentin and sealer and increases the bond strength values in comparison with dentin without treatment [20].

In general, it was evidenced that AH26 presents the highest bond strength among sealers [6,7,8,9]. The use of AH26 in this study was based on its molecular bonding mechanism. The ability of epoxy-resin-based AH26 to react with any exposed amino groups in collagen, forming covalent bonds between resin and collagen when the epoxide ring opens, represents an adhesion mechanism that is directly affected by the adhesion substrate, i.e., the exposed collagen on dentin surface [1]. This leads to the assumption that separating the highly cross-linked collagen into a sporadic network of distinct fibrils in a dentin matrix using a solvent like DMSO increases the quality and quantity of the covalent bonds of the epoxide ring with the exposed amino groups of dentin collagen, leading to enhanced adhesion. So far, substantial evidence has been provided that favors AH26 adhesion to dentin, even without this additional mechanism. It was documented that AH26 displays improved bond strength values when compared with other types of endodontic sealers [6,7,8,9]. Regarding shear bond strength, Salman et al. concluded that AH26 provided better results than Diaket and Ketac-Endo. Most importantly, besides the bonding system, the pre-treatment of dentin with a chelating agent increased the adhesive potential of the AH26 sealer [21].

So far, the positive effect of DMSO on dentin bonding was elucidated by many studies. Mehtala et al. found that DMSO pre-treatment on wet dentin improved adhesive bonding by affecting the exposure of dentin’s apparent collagen fibrils [14]. This observation is in agreement with other studies showing that DMSO irrigation improves the quality of the collagen–resin biopolymer at the bonded interface by reducing the extent of the exposed highly cross-linked collagen matrix, suggesting that 50% DMSO could be a feasible alternative to improving the resin–dentin bond quality, especially for water-based etch-and-rinse adhesive systems [13,14]. However, all relevant studies using DMSO compared the effect of DMSO on the adhesion of dentin bonding agents. Since this solvent is capable of expanding the collagen matrix, solvates adhesive components, infiltrates dentinal collagen, has low toxicity, and improves both immediate and long-term bond strength, this makes DMSO a possible new solvent for improving the adhesive potential of epoxy resin sealers to dentin. In this study, the interaction of DMSO-treated dentin with AH26 was evaluated. Unlike the previously published reports that favored the use of this solvent with bonding agents, this study revealed that dentin pretreatment with DMSO did not have a significant effect on the AH26 adhesive properties. Consequently, the chemical and micromechanical adhesion of AH26 sealer to dentin was not affected. This could be attributed to the difference in the bonding mechanism between the dentin bonding agents and epoxy resin sealers. Perhaps the chemical composition of the specific monomers in self-etched adhesives and the microstructure of the desired collagen resin biopolymer present substantial differences when compared with the sealer’s monomers and, more specifically, the epoxy ring connections with dentin’s amino groups.

Regarding the failure mode, the microscopic examination of fractured surfaces revealed that adhesive failure was the main bond failure. Since the sealer was the only obturation material involved, a cohesive mode of failure was less likely to prevail. It was shown that the weaker bond of epoxy resin sealers with gutta-percha than with dentin leads to a cohesive mode of failure when using the push-out method for bond strength evaluation [22,23,24]. Consequently, the absence of a weaker gutta-percha/sealer bond, along with the type of bond strength evaluation method, could affect the failure mode prevalence. However, other studies that also used the push-out test provided evidence of an adhesive failure mode of epoxy resin sealers [25,26].

Previous reports comparing the shear bond strength test (SBS) with the push-out test concluded that the shear bond strength evaluation method seems to be a more simple, effective, and reproducible method, and is suitable for testing gutta-percha and dentin samples in a similar manner [27]. It provides homogenous results with a significantly low variation of bond strength [28]. It is considered to be a more appropriate method for materials with greater plasticity, such as gutta-percha, where the push-out method is contraindicated. When comparing the bond strength of different root-canal-filling materials, the easier standardization of the specimen by using flat surfaces represents another advantage of the shear bond strength test. In this study, flat surfaces were used to estimate the shear bond strength of AH26 to coronal dentin in order to reduce sample heterogeneity. Considering that root dentin is not uniform and the walls of the root canal may present major irregularities, coronal dentin was selected rather than root dentin, which is traditionally used for push-out testing. In addition, the gradual decrease in the number of dentinal tubules from the coronal to the apical part of dentin is another important parameter that could affect the homogeneity of the tested specimen [29].

Additionally, some unexplored variables could alter the bond strength, such as fluid contamination. Charuphan Oonsombat et al. found that blood contamination during any stage of bonding with a self-etch primer significantly decreased the shear bond strength of orthodontic brackets to enamel [30]. Another factor that affects bond strength is fluoride application of the bonding process on the shear bond strength. Fluoride application before both conditioning and bonding results in significantly lowers bond strength values [31]. These variables should be tested in future laboratory and clinical trials.

Many studies investigated the effect of different dentine pre-treatment agents on the adhesion of endodontic sealers. Saleh et al. examined the adhesion of different root–canal sealers, including Grossman’s sealer, Apexit, Ketac-Endo, AH Plus, RoekoSeal Au-tomix, or RoekoSeal Automix, to dentine treated with phosphoric acid 37%, EDTA 17%, or citric acid 25%. This study showed that pre-treatment with EDTA had no effect or produced weaker bonds than controls and phosphoric/citric acid pretreatment increased the adhesion only for one type of sealer (Grossman) [7]. Another study evaluated the effect of MTAD and EDTA as a final rinse on the shear bond strength of Kerr, Apexit, and AH plus sealer. It was concluded that AH plus showed superior bond strength among the sealers when EDTA was used, whereas using MTAD as a final rinse affected the bond strength of AH plus and Apexit [32]. These results provide additional evidence supporting the claim that different endodontic sealer types require different dentine pre-treatments for effective adhesion [7].

Despite the fact that shear bond strength may offer reproducible and valid results of homogenous test groups by limiting any physical morphological or anatomical variations of the specimen, two of the main disadvantages of this method include the difficulty of narrowly aligning the device of shear-loading with the adhesive interface and the apparent difficulties in simulating real clinical conditions, i.e., the application of the load is not perpendicular to the dentin tubules, which stimulates the real forces acting inside the root canal [33]. Another limitation of this study was that the only sealer used was AH26. The use of this sealer was based on the type of its molecular bonding mechanism and the presentation of the highest bond strength relative to other sealers. More studies should be performed to evaluate possible interactions between DMSO and dentin to improve the bond strength of endodontic sealers, including AH26. The outcomes of this study constitute the first step toward establishing the relationship between DMSO and endodontic sealers and should be the trigger for relevant future studies that modify the present experimental protocol. Future studies should use DMSO in different concentrations for different durations of DMSO application since these two factors have major effects on the activity of this solvent.

## 5. Conclusions

From the results of this study, it was concluded that DMSO had no substantial effect on the bond strength of AH26 root sealer to dentin. Further studies should investigate the optimal use of DMSO to enhance the bond strength to dentin in an effort to improve the adhesion and seal of obturation materials.

## Figures and Tables

**Table 1:** Materials used in this study.

Materials	Manufacturer	Composition
AH26 silver free (LOT: 1809000118)	Dentsply, Konstanz, Switzerland	Powder: bismuth oxide, methenamine Gel: epoxy resin
DMSO (LOT: E2119)	Santa Cruz Biotechnology, Heidelberg, Germany	C_2_H_2_OS

**Table 2:** Dentin treatment and materials.

Group	Dentin Treatment	Materials
A	3 mL EDTA 17%, 2 min Drying with air spray, 10 s	AH26
B	3 mL EDTA 17%, 2 min Rinse with distilled water, 10 s Drying with air spray, 10 s DMSO, 60 s Gently air dry, 5 s AH26	DMSO AH26

**Table 3:** Descriptive statistics of the involved variables.

Variable	Mean	Std Deviation	Minimum	Maximum
Group A	0.8924	0.3753	0.2256	1.7162
Group B	0.8927	0.4164	0.2157	2.0299

**Table 4:** Paired samples *t*-test results.

Pair of Variables	Mean Difference	Standard Error	t-Statistic	*p*-Value
Group A–Group B	−0.00028	0.07119	−0.004	0.997

**Table 5:** Fracture models of the materials.

Material	Adhesive Failure	Cohesive Failure	Mixed Mode Failure	Confidence Interval
AH26	27 (77.1%)	5 (14.3%)	3 (8.6%)	95%
AH26 + DMSO	25 (71.5%)	6 (17.1%)	4 (11.4%)	95%
